# What is chiropractic?

**DOI:** 10.1186/s12998-017-0163-x

**Published:** 2017-11-02

**Authors:** Jan Hartvigsen, Simon French

**Affiliations:** 1 Department of Sports Science and Clinical Biomechanics, University of Southern Denmark, Campusvej 55, 5230 Odense M, Denmark; 2 Nordic Institute of Chiropractic and Clinical Biomechanics, Campusvej 55, 5230 Odense M, Denmark; 3 School of Rehabilitation Therapy, Faculty of Health Sciences, Queens University, 99 University Ave, Kingston, ON K7L 3N6 Canada; 4 Department of Chiropractic, Macquarie University, Sydney, NSW 2109 Australia

**Keywords:** Chiropractic, Editorial, Health policy

## Abstract

While in some jurisdictions chiropractic is fully integrated in public and insurance funded health care systems, in others it is outside and considered as complementary or alternative health care. There is a paucity of data and rigorous scientific studies regarding most aspects of chiropractic practice although research activity has been increasing in recent years. We call for papers for a thematic series in *Chiropractic and Manual Therapies* that can help define chiropractic better to stakeholders inside and outside the profession under the theme *What is Chiropractic?*

## Background

In 2002, Meeker and Haldeman wrote that “*In today’s dynamic health care milieu, chiropractic stands at the crossroads of mainstream and alternative medicine*” [1]. Fifteen years later the global identity and place for chiropractic in healthcare is still unresolved. From within chiropractic, one end of the spectrum subscribes to “*a philosophy of neo-vitalism”* and “*a neurologically-centered model of subluxation*” [2], while the other end promotes that “*chiropractic education should be ……… founded on the principles of evidence-based care”* and “*the teaching of vertebral subluxation complex as a vitalistic construct that claims that it is the cause of disease is unsupported by evidence”* [3]. In some parts of the world chiropractors are highly integrated in insurance or government funded healthcare programs and educated in government funded universities, whereas in other parts chiropractors are educated in private schools and practice as complementary and alternative practitioners completely outside of mainstream healthcare. Further, some academic authorities outside of chiropractic dismiss chiropractic as a legitimate health care profession [4], whereas others publically endorse the role of chiropractors in the management of specific health problems [5], adding to the confusion about the role the chiropractic profession plays.

The description and assessment of any healthcare profession should be based on rigorous data and scientific scrutiny rather than preconceived opinions. Although research dealing with the activities and effectiveness of the chiropractic profession is still relatively scarce compared to other musculoskeletal-focused professions [6], research productivity is increasing. Our informal search in Pubmed using the search terms “chiropractic OR chiropractor” reveals a 54% increase in publications between 2002 and 2016 (216 versus 401 publications). Hence, we have a better understanding than ever before of the education of chiropractors [7, 8], the types of therapies used by chiropractors [9–11], the conditions treated by chiropractors [10–13], the effectiveness [14–16] and cost-effectiveness of chiropractic care [17, 18], safety and side effects of chiropractic treatment [19, 20], and settings where chiropractors function alone or in collaboration with other healthcare providers [21–23]. In spite of this, there is still not a clear picture of what comprises the chiropractic profession, and how chiropractors contribute to the health of individual patients and populations.

It is therefore pertinent and timely to launch a thematic series of papers in *Chiropractic & Manual Therapies* that will address the broad overarching question “What is chiropractic?”. We hope these papers will provide a critical overview of the current state of the chiropractic profession from a global perspective. Individual articles will address fundamental issues about the chiropractic profession, including descriptions of: how the profession is placed in different countries and settings; the history of the profession in some countries; chiropractic education around the world; the profile of patients who seek chiropractic care; the types of care chiropractors provide; outcomes of chiropractic care; and other aspects of chiropractors, their practice, evidence base, and chiropractic care. We hope this thematic series will contribute to a clearer picture of chiropractic including an enlightened discussion, both within and outside of the chiropractic profession, of the role chiropractors play globally, and how they contribute to the health and well-being of the millions of people who seek their care every year.

## Conclusions

There is a paucity of data and rigorous scientific studies regarding most aspects of chiropractic and chiropractic practice. We welcome all types of submissions to this thematic series from researchers of all backgrounds and locations.
